# Short-term exposure to a high-humidity environment triggers intestinal inflammation via AQP3

**DOI:** 10.3389/fimmu.2025.1563602

**Published:** 2025-06-18

**Authors:** Jian Song, Xinhua Huang, Yi Luo, Mengjun Li, Yulin Ouyang, Wanli Liu, Hudan Pan, Huanhuan Luo

**Affiliations:** ^1^ State Key Laboratory of Traditional Chinese Medicine Syndrome, Guangzhou University of Chinese Medicine, Guangzhou, China; ^2^ Chinese Medicine Guangdong Laboratory, Guangdong-Macao In-Depth Cooperation Zone in Hengqin, Zhuhai, China; ^3^ School of Basic Medical Sciences, Guangzhou University of Chinese Medicine, Guangzhou, China; ^4^ School of Pharmaceutics, Guangzhou University of Chinese Medicine, Guangzhou, China; ^5^ State Key Laboratory of Dampness Syndrome of Chinese Medicine, The Second Affiliated Hospital of Guangzhou University of Chinese Medicine, Guangzhou, China

**Keywords:** AQP3, high-humidity environment, intestine, inflammation, PPAR-γ

## Abstract

**Background:**

With the increasing greenhouse effect, there is growing concern about the correlation between the humid environment and the incidence of various diseases. A high-humidity environment may cause intestinal inflammation through bacterial colonization or contamination of water. Aquaporin-3 (AQP3) plays an important role in maintaining intestinal water transport, permeability, fluid secretion, and absorption homeostasis. This paper explored the effects of short-term exposure to a high-humidity environment on intestinal health.

**Methods:**

To explore the effects of a high-humidity environment on the intestine, we kept wild-type mice and Aqp3 knockout (*Aqp3^-/-^*) mice in an artificial climatic box with 90(± 5) % humidity setting for a fortnight and recorded their body weights, food intake, water intake, and fur changes during the experiment. On the fourteenth day, colon tissues were collected to detect the expression of intestinal inflammatory factors, glutathione (GSH), malondialdehyde (MDA), water evaporator proteins (AQPs), and intestinal pathological changes by polymerase chain reaction (PCR), Western blotting analysis, and histopathological analysis.

**Results:**

The results showed that mice with short-term exposure to a high-humidity environment showed a significant increase in the expression of AQP3 and a significant decrease in the expression of AQP4 in the colon, and the TLR4/NF-κb/IL-6 pathway was activated. In *Aqp3^-/-^* mice, their colonic GSH expression was increased, MDA expression was decreased, and intestinal TLR4/NF-κb/IL-6 expressions were also decreased.

**Conclusions:**

This study demonstrated the high-humidity environment induces an intestinal inflammatory response through AQP3, providing persuasive evidence for the pathogenesis of environmentally related diseases.

## Graphical Abstract

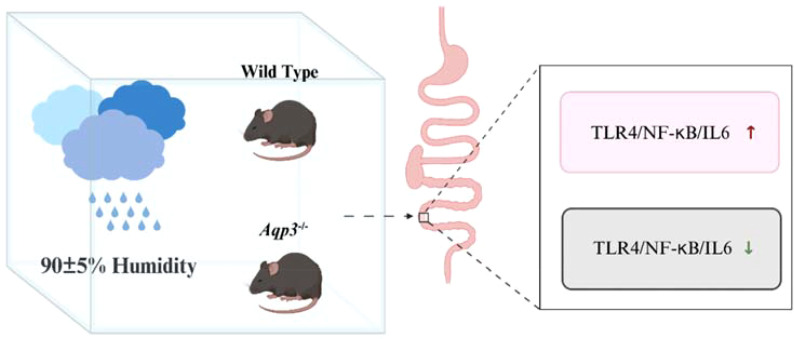

## Highlights

- High-humidity environment impacts intestinal Aquaporins.
- High-humidity environment enhances AQP3 expression.

## 1 Introduction

Under current greenhouse gas emissions, climatologists predict there will be a warmer and more humid Earth’s climate in the upcoming century (1, 2). Wherein, increasing humidity may directly or indirectly trigger several diseases (1). According to environmental epidemiology, humidity is associated with all-cause mortality and morbidity, including cardiovascular, pulmonary, and gastrointestinal system diseases (1). One research proposed it is necessary to study the health effects of temperature and humidity individually to recognize the negative/positive influence of humidity-related mortality in hot and dry or warm and humid climates (3). In addition to temperature, atmospheric humidity is a key climate component that might affect human health (4). A study found that relative humidity (RH) above 70% causes a significant impact on human response, amplified with increasing temperatures (5). Therefore, it is crucial to study the impacts of high humidity on health.

The alimentary canal is directly connected to the external world and vulnerable to external environmental factors (6). A study found that low-temperature and high-humidity environments caused intestinal inflammation in mice (7). Our previous studies found that high temperature and humidity induced intestinal inflammation in mice (8, 9). In addition, high-humidity environments could increase the risk of intestinal infectious diseases (10). However, more research is still needed to focus on the effects of high-humidity environments on the intestinal tract.

Humidity is one of the common external environmental factors affecting body’s water metabolism (6). For example, a high-humidity (90 ± 2%) environment increases blood urea nitrogen and antidiuretic hormone secretion to affect the colon and lead to water metabolism disorders (11). Clinical studies have observed that high humidity during summer and autumn induces diarrheal diseases (12, 13). Aquaporins (AQPs) are a family of membrane proteins that efficiently and selectively facilitate the transport of water across biological membranes (14–16). They are essential for maintaining water homeostasis in cells and tissues. The wide distribution of AQPs in the human intestine implies that they are probably important in channel-mediated water transport, intestinal permeability, and fluid secretion/absorption (14). In addition, AQPs are involved in the release of inflammatory factors and mediators (15). Among them, Aquaporin-3 (AQP3)is a major water channel protein expressed in the intestine and plays important roles in gut physiology and pathophysiology due to its permeability to water, glycerol, and hydrogen peroxide (17). Alterations of AQP3s in the gut may be associated with a variety of intestinal disorders such as inflammatory bowel disease, diarrhea, intestinal barrier injury, irritable bowel syndrome, intestinal oxidative stress, and autophagy (18).

Thus, the high-humidity environment can be reasonably hypothesized to affect AQPs in the bowel and may cause an inflammatory response. In this study, an artificial climate box was used to simulate a high-humidity environment and to explore its effects on the colon as well as on colonic AQPs.

## 2 Materials and methods

### 2.1 Animal experiments and ethical approval

Male-specific pathogen-free (SPF) C57BL/6N mice and AQP3-knockout (*Aqp3^-/-^*) mice weighing 20 ± 2g were purchased from Guangdong Medical Experimental Animal Center (China). Mice were housed in SPF facilities with free access to food and water in a controlled environment (22 ± 1°C, 55 ± 5% relative humidity, 12h light/dark cycle). No experiment was performed until all the mice had acclimated for seven days. This study was performed under the supervision and assessment of the Laboratory Animal Ethics and Welfare Committee (AEWC) of Zhongshan Hospital of Traditional Chinese Medicine (No. AEWC-2023027). All experimental procedures were performed by the recommendations of the National Institutes of Health Guide for the Care and Use of Laboratory Animals [National Research Council, Guide for the Care and Use of Laboratory Animals (2011).].

To investigate the effect of a high-humidity environment on mice, sixteen C57BL/6N mice were randomly and equally divided into two groups, the normal environment control group (NC) and the high-humidity environment group (HH).

In the second part of the experiment, sixteen C57BL/6N mice as well as sixteen *Aqp3^-/-^* mice were randomly and equally assigned to 4 groups: the normal environment control group (NC), the high-humidity environment group (HH), the normal environment knockout group (Ko-NC) and the high-humidity environment knockout group (Ko-HH).

Using an artificial climate box (model: LAC-475-N, Shanghai Longyue Instruments Co., Ltd, China) to simulate the high-humidity environment. Mice in both HH and Ko-HH groups were exposed to 22 ± 2°C, 90 ± 5% relative humidity, and a 12h light/dark cycle for 14 days. Mice in the NC and Ko-NC groups were housed in the controlled environment as the normal environment for 14 days. Considering the rapid growth of bacteria in the high-humidity environment, food, and drinking water are sterilized and renewed daily to avoid contamination. All mice had free access to water and food during the experiment.

### 2.2 Sample collection

The colon tissues were rapidly removed and divided into two portions. One portion of the sample was homogenized with sterile phosphate buffered saline (PBS) for the detection of 2 oxidative stress-related indexes (glutathione (GSH) and malondialdehyde (MDA)). The other portion was immediately placed in liquid nitrogen and stored at -80°C for subsequent mRNA expression analysis and western blotting assay.

### 2.3 Mouse colon histopathological analysis

For colon histological studies, mice were euthanized and colon tissues were fixed with neutral buffered formalin 10% and embedded in paraffin. Tissue sections were prepared and stained with Hematoxylin and Eosin(H&E) (19).

### 2.4 RNA isolation and quantitative reverse transcriptase polymerase chain reaction

Total RNA was extracted from mouse colons using TRIzol reagent (TermoFisher Scientific, Shanghai, China), and reverse transcribed to cDNA using the PrimeScript RT Master Mix (Takara Biomedical Technology Co., Ltd, China), and quantified via real-time PCR analysis using an ABI 7500 real-time PCR system (Applied Biosystems Inc., CA, USA). PCR was performed in triplicate using SYBR Green (Takara Biomedical Technology Co., Ltd, China). cDNA was amplified for 40 cycles using a preset cycling program that included the generation of a melting curve. Thermocycling conditions were as follows (CFX CONNECT, Bio-Rad, USA): (1) 50°C for 2min (activation of AmpErase UNG); (2) 95°C for 10min; (3) 95°C for 15s (denaturation) and 60°C for 1min (annealing/extension) for 40 cycles. The relative expression levels of each gene were normalized against the expression of GAPDH (20). Sequences of the primers used for analysis were listed in **Supplementary Table 1**.

### 2.5 Western blotting assay

To extract total protein, colon tissues were lysed in RIPA buffer supplemented with 1% protease inhibitor cocktail and centrifuged at 12,000 rpm and 4°C for 15min. Protein concentration was determined using the BCA Protein Assay Kit (TermoFisher Scientific, Shanghai, China). An equal amount of protein (20 μg) was loaded on 10% sodium dodecyl sulfate (SDS)–polyacrylamide gels for separation. The separated proteins were transferred to a polyvinylidene difluoride (PVDF) membrane and blocked with 5% skim milk in TBST for 2h at 25°C. Subsequently, the membrane was incubated with primary antibodies Aquaporin-3(AQP3) (1:1000), Aquaporin-4(AQP4) (1:1000), Interleukin-6 (IL-6) (1:1500), Nuclear factor kappa-B (NF-κB) p65(1:1000), Peroxisome proliferator-activated receptor γ (PPAR-γ) (1:1500), GAPDH (1:10000) (Proteintech Group, Inc. USA) at 4°C over-night. The following day, the membrane was washed with TBST and incubated with secondary antibodies (1: 2500 dilution) for 1 h at 25°C. Images were captured using a gel imaging system according to the manufacturer’s instructions (5200 Multi, Tanon, China), and GAPDH was used as the loading control (20).

### 2.6 The detection of MDA and GSH

The obtained tissue homogenates of the colon were used to detect GSH and MDA contents. A GSH assay kit (Microplate method, number: A006-2-1) and MDA assay kit (TBA method, number: A003-1-2) were used for these measurements. Both kits were purchased from Nanjing Jiancheng Bioengineering Institute (Nanjing, China). All the experimental operations were carried out following the manufacturer’s instructions (21, 22).

### 2.7 Statistical analysis

For two experimental groups, *t-test* or *nonparametric test* was used to compare the differences. *One-way ANOVA* or *Kruskal-Wallis* multi-comparisons test was used to compare the differences across the four experimental groups and continued to use *Tukey’s* method or *Bonferroni correction* for further analysis (GraphPad Prism 10.1.2 software, Inc., San Diego, CA, USA). Data were presented as mean ± standard deviation (SD) (n ≥4) for each group. P values < 0.05 were considered significant (*P < 0.05; **P < 0.01; ***P < 0.001; ****P < 0.0001).

## 3 Results

### 3.1 The high-humidity environment affected the colonic AQP3 and triggered an inflammatory response

**Figure 1:**
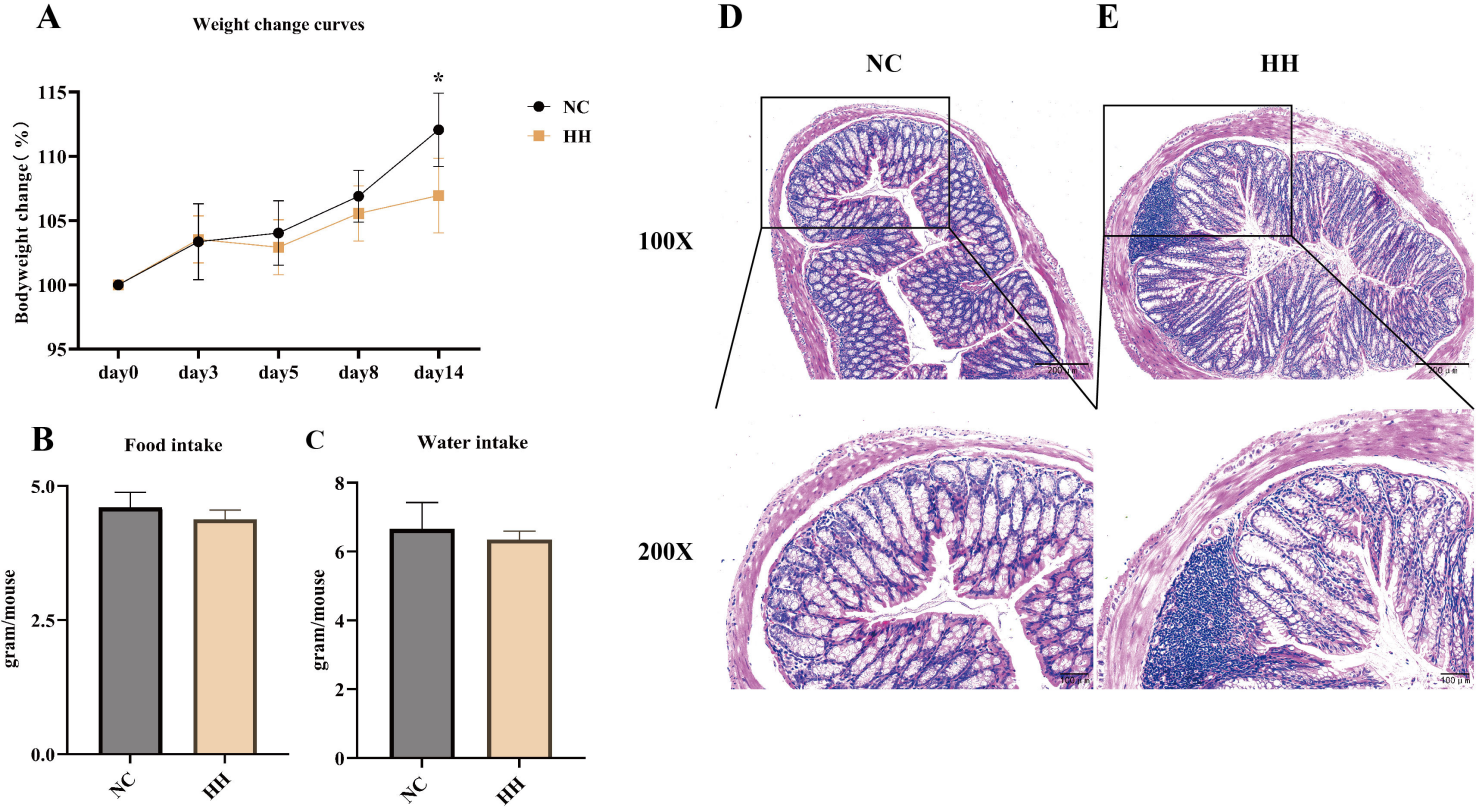
The body weight, food and water intake, and pathological changes of the colon were altered in mice after 14 days of being exposed to the high-humidity environment. **(A)** Body weight change curves of mice (n = 8). **(B, C)** Average food intake and water intake of mice on day 14 (Each group of mice was housed in two cages, so no statistical data could be analyzed) (n = 8 mice). **(D, E)** Histological changes in the distal colon of the NC group and the HH group mice stained with hematoxylin and eosin (H&E), with 100x and 200x magnification. Data were analyzed by unpaired t-test and expressed as the mean ± SD. Compared with NC: * *P*<0.05.

To investigate the effects of the high-humidity environment, mice were placed to live for 14 days. No mice deaths occurred during the experiment. Throughout the experiment, the body weights of all mice increased gradually. At the end of the experiment, there was a significant difference with 6% body weight increase in the HH group and 12% in the NC group (*P*<0.05) (**Figure 1A**). During the experiment, mice in the NC group were very active and had sleek coats. In contrast, mice in the HH group gradually showed inactivity, unkempt fur, and lower food and water intake than those in the NC group (**Figures 1B, C**). The results of Hematoxylin-eosin staining of colon sections were shown in **Figures 1D, E**. Under the light microscope, the NC group had a normal colonic histological structure with tightly connected epithelial cells, intact crypt structure, and no inflammatory cell infiltration. No obvious pathological change was seen in the HH group.

**Figure 2:**
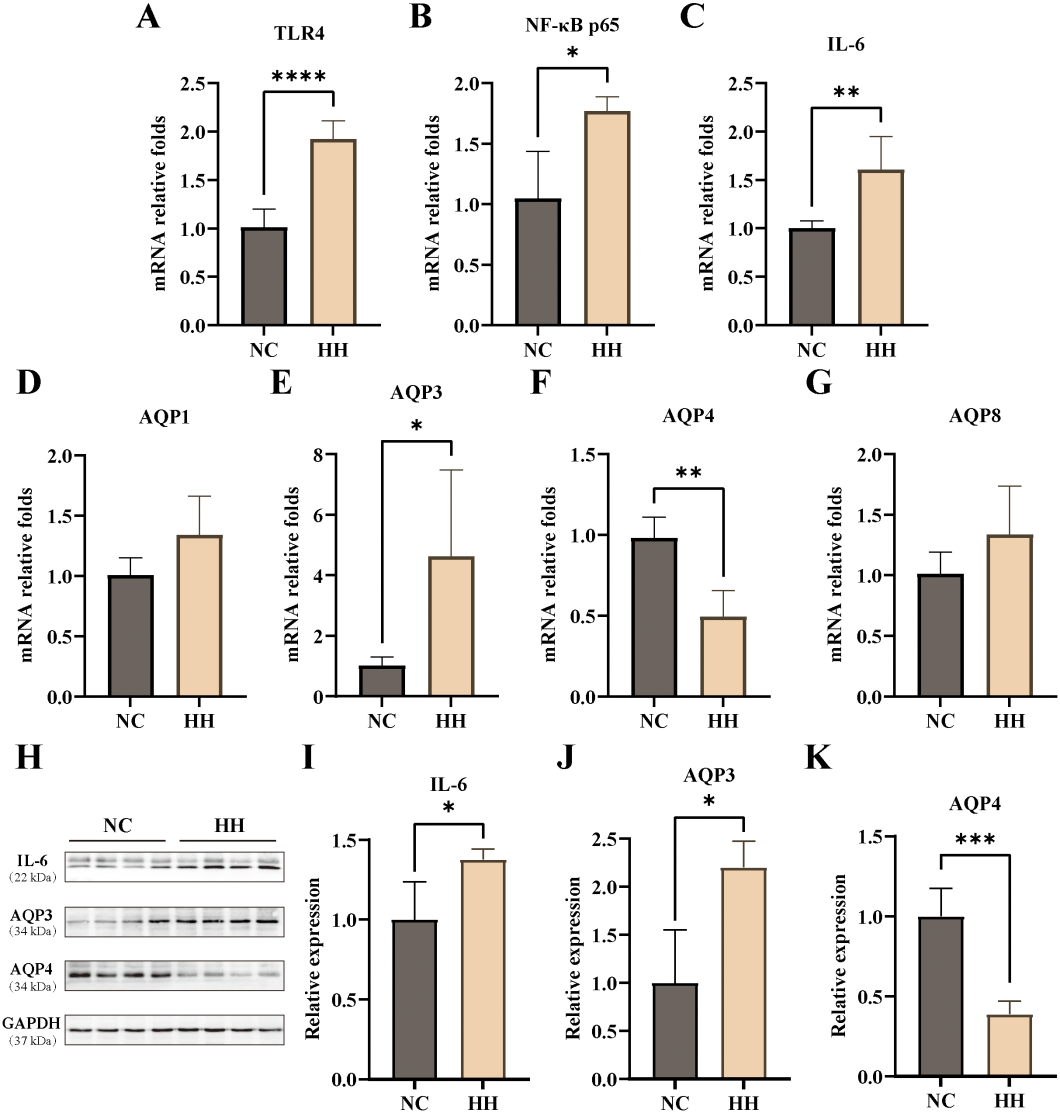
Expression levels of AQPs and inflammatory factors in the mouse colon. **(A-G)** The mRNA relative expression levels of TLR4, NF-κB, IL-6, AQP1, AQP3, AQP4, AQP8 (n=4-6). **(H-K)** The protein expressions of IL-6 **(H, I)**, AQP3 **(H, J)**, and AQP4 **(H, K)** were analyzed via Western blotting and then relatively quantitatively measured (n=4). Data were analyzed by unpaired t-test and expressed as mean ± SD. Compared with NC: * *P*<0.05; **P<0.01; ***P<0.001; ****P<0.0001.

To further explore the effects of the high-humidity environment on the colon, we assayed the aquaporins and inflammatory factors. The results showed that the mRNA expression levels of TLR4, NF-κB p65, and IL-6 in the HH group were elevated and significantly different from those in the NC group (*P*<0.0001, *P*<0.05, *P*<0.01) (**Figures 2A–C**). Compared with the NC group, the mRNA expression levels of Aquaporin-1 (AQP1), AQP3, and Aquaporin-8 (AQP8) were raised in the HH group, with a significant difference in AQP3 expressions (*P*<0.05). In contrast, the expression level of AQP4 was decreased in the HH group, which was significantly different from the NC group (*P*<0.01) (**Figures 2D–G**). Furthermore, the western blotting assay showed that the relative expression levels of IL-6, AQP3, and AQP4 proteins were consistent with the mRNA expression levels, and were significantly different from those of the NC group (*P*<0.05, *P*<0.05, *P*<0.001) (**Figures 2H–K**). According to our group’s previous studies, high temperature and humidity caused intestinal inflammatory factors elevation and minimal enteritis in mice (8). And, intestinal AQP3 was upregulated in mice with Chinese dampness-heat syndrome diarrhea by improper diet combined with high temperature and humidity environments (23). It has been suggested that AQP3 may be involved in intestinal inflammation (17). In contrast, AQP4 knockdown had little effect on the important functions of the colon (24). The results of this study showed that the expression of AQP3 was consistent with NF-κB, TLR4, and IL-6. Therefore, we hypothesized that the high-humidity environment induces intestinal inflammatory response by affecting intestinal AQP3 and conducted the next experiments by knocking down AQP3.

### 3.2 The high-humidity environment facilitated an inflammatory response through AQP3

**Figure 3:**
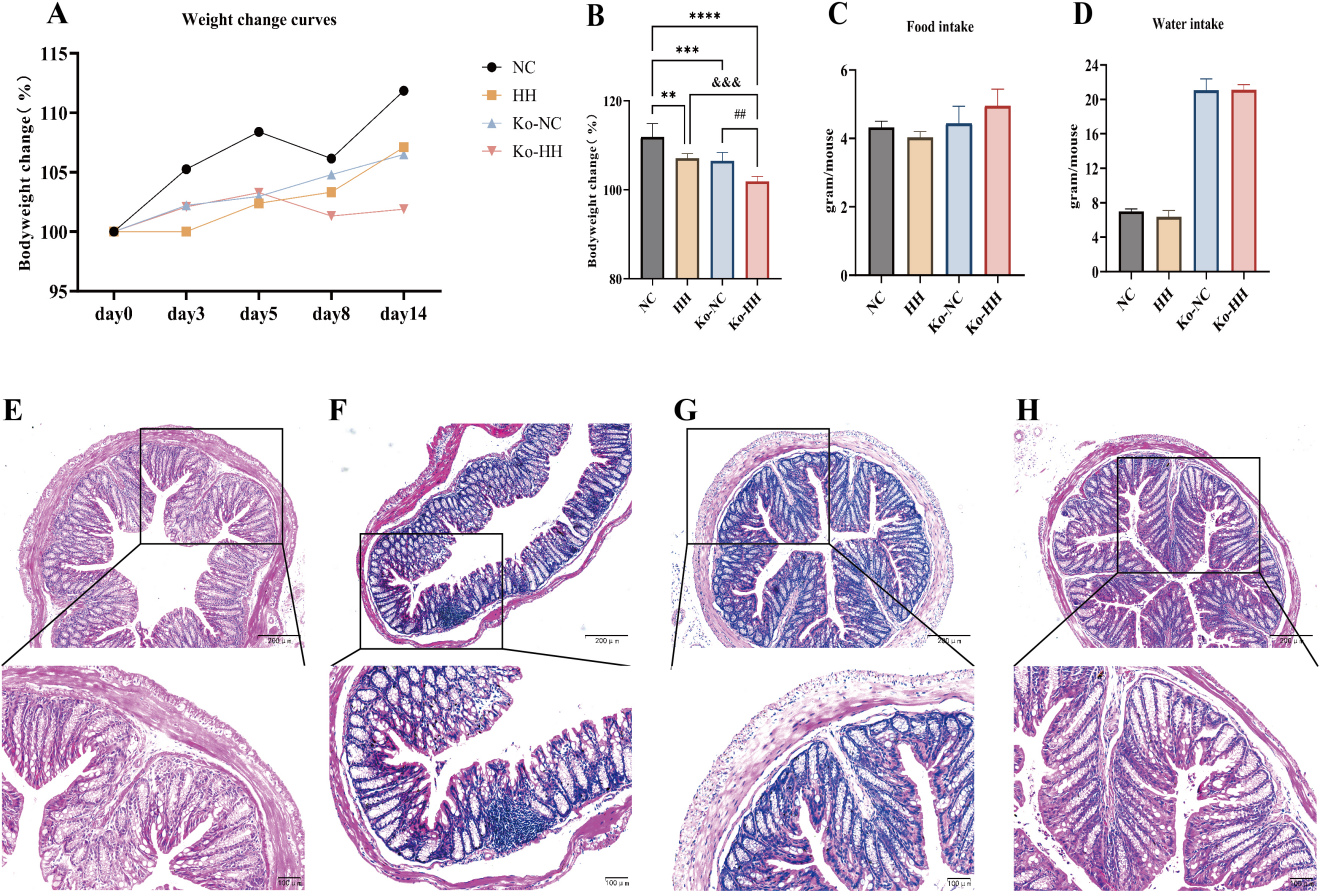
Alterations in body weight, food and water intake, and colonic pathology in both mice and *Aqp3^-/-^* mice after 14 days of exposure to HH. **(A)** Body weight change curves for each group of mice (n=6). **(B)** Body weight changes in each group of mice on day 14 (n=6). **(C, D)** Average food and water intake in each group of mice on day 14 (Each group of mice was housed in two cages, so no statistical data could be analyzed). **(E-H)** Histological changes in the distal colon of each group of mice stained with H&E, with 100x and 200x magnification. Data were expressed as the mean ± SD. Compared with NC: **P<0.01; ***P<0.001; ****P<0.0001. Compared with Ko-NC: ## *P*<0.01. Compared with HH: &&& *P*<0.001. One-way ANOVA followed by Tukey’s multiple comparisons test **(B)**.

To verify our hypothesis, we carried out experiments with *Aqp3^-/-^* mice. The body weights of mice in all groups increased progressively throughout the experiment. Among them, the mice in the Ko-HH group showed the slowest growth in body weight. At the end of the experiment, mice in the NC group grew by 11.8%, those in the HH group by 7.1%, in the Ko-NC group by 6.5%, and in the Ko-HH group by 1.8% (**Figures 3A, B**). All groups showed statistically significant differences comparing each other, except for the comparison between the HH group and the Ko-NC group. During the experiment, both NC and Ko-NC mice were active and had smooth fur. In the high-humidity environment, both HH and Ko-HH mice showed inactivity and messy fur.

The results showed that the food intake and water intake of *Aqp3^-/-^* mice were higher than those of wild-type mice, whether in a normal environment or a high-humidity environment (**Figures 3C, D**). The higher water intake of *Aqp3^-/-^* mice was speculated to be due to their characteristics of polyuria (25, 26). Among the wild-type mice, the mice in the HH group showed lower food and water intake than those in the NC group. However, *Aqp3^-/-^* mice showed a slight increase in food intake in the high-humidity environment compared to mice in the normal environment.

The results of Hematoxylin-eosin staining of colon sections were shown in **Figures 3E–H**. Under the light microscope, no obvious pathological changes were seen in the colonic tissues of the four groups of mice.

**Figure 4:**
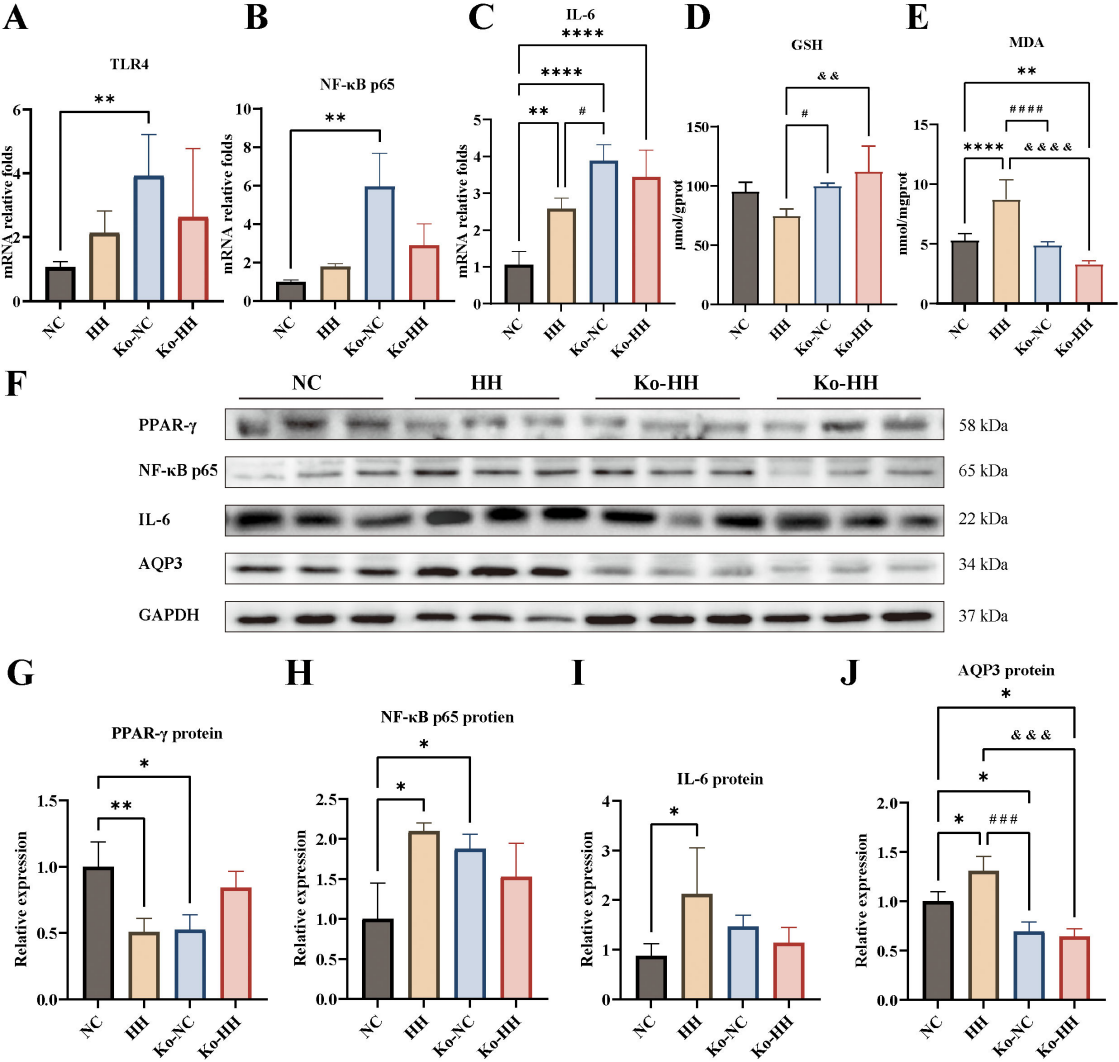
Expression levels of AQP3, GSH, MDA and inflammatory factors from the colon of mice in each group. **(A-C)** The mRNA relative expression levels of TLR4, NF-κB p65, IL-6 (n=4-5). **(D, E)** GSH and MDA in colon (n=5). **(F-J)** The protein expressions of PPAR-γ **(F, G)**, NF-κB p65 **(F, H)**, IL-6 **(F, I)** AQP3 **(F, J)** were analyzed via Western blotting and then relatively quantitatively measured (n=3). Data were expressed as mean ± SD. Compared with NC: *P<0.05; **P<0.01; ****P<0.0001. Compared with Ko-NC: #*P*<0.05; ###*P*<0.001; ####*P*<0.0001. Compared with HH: &&&*P*<0.001; &&&&*P*<0.0001. One-way ANOVA followed by Tukey’s multiple comparisons test **(A, C-E, G-J)** or Kruskal-Wallis test **(B)**.

Similarly, the mouse colon was assayed for AQP3, inflammatory factors, Malondialdehyde (MDA), and Glutathione (GSH). The results demonstrated that TLR4, NF-κB p65, and IL-6 were higher in the HH group of mice than in the NC group, with a significant difference in IL-6 (*P*<0.01) (**Figures 4A–C**). In *Aqp3^-/-^* mice, inflammatory factors were all lower in the Ko-HH group of mice than in the Ko-NC group (no significant difference). In the normal environment, the mRNA expression levels of inflammatory factors were higher in the Ko-NC group compared with the NC group and statistically different (*P*<0.01, *P*<0.01, *P*<0.0001) (**Figures 4A–C**). Compared with the HH group, the mRNA expression levels of inflammatory factors were higher in the Ko-NC group, with a significant difference in IL-6 (*P*<0.05) (**Figure 4C**). In the high-humidity environment, higher levels of inflammatory factor mRNA expression were found in the Ko-HH group than in the HH group (no significant difference). In comparison with the NC group, the Ko-HH group showed higher mRNA expression levels of inflammatory factors, of which IL-6 expression levels were significantly different (*P*<0.0001) (**Figure 4C**).

Oxidative damage represents one of the main triggers of the inflammatory process. Subsequently, the levels of GSH and MDA in the colon were measured with kits (**Figures 4D, E**). The results demonstrated that GSH content was reduced in the HH group compared to the NC group and increased to varying degrees in the Ko-NC and Ko-HH groups (no significant difference). Compared with the Ko-NC group, the GSH content was higher in the Ko-HH (no significant difference) and lower in the HH group (*P*<0.05). In the high-humidity environment, GSH content was more in the Ko-HH group than in the HH group (*P*<0.01). In the experiment of detecting the content of MDA, it was found that the HH group had the highest content and there was a significant difference in comparison with all the other groups (*P*<0.0001, *P*<0.0001, *P*<0.0001). There was no significant difference in the content of GSH in the Ko-NC group and the NC group. In other group comparisons, the Ko-HH group had the lowest content, of which there was a significant difference in comparison with the NC group (*P*<0.01). Further, the PPAR-γ protein expression levels were lower and statistically different in both the HH and Ko-NC groups compared with the NC group (*P*<0.01, *P*<0.05) (**Figures 4F, G**). The PPAR-γ protein level in the Ko-HH group, by contrast, was higher than that in the HH and Ko-NC groups.

In the inflammatory response, it was found that in contrast to the NC group, the protein levels of the remaining three groups showed different elevations, wherein the HH group was the highest (**Figures 4F, H, I**). The protein expression levels of NF-κB p65 as well as IL-6 were increased in the HH group in comparison with the NC group and showed statistically significant differences (*P*<0.05, *P*<0.05). The NF-κB p65 expression level was higher in the Ko-NC group than in the NC group (*P*<0.05). In the Ko-HH, meanwhile, the protein expression levels of NF-κB p65 as well as IL-6 were lower than those in the HH and Ko-NC groups. In wild-type mice, the high-humidity environment promoted the protein expression of AQP3 (*P*<0.05) (**Figures 4F, J**). Regardless of the environment, there was no significant difference in AQP3 protein expression between the Ko-NC and Ko-HH groups, and both were lower than the NC (*P*<0.05, *P*<0.05) and HH groups (*P*<0.001, *P*<0.001).

## 4 Discussion

Increasing environmental humidity may be harmful to human health such as the gastrointestinal tract (1). As such, knowledge of the changes occurring in the intestinal tract in a high-humidity environment would be available to benefit human health. In this study, an artificial climate box was used to simulate a high-humidity environment to investigate the effects of a short-term high-humidity environment on the colon. In our previous studies, high temperature and humidity environment resulted in decreased appetite in mice (9, 27). This study similarly observed that the high-humidity environment resulted in decreased appetite, slow weight gain and decreased water intake in mice. *Aqp3* ^-/-^ mice also showed a slow increase in body weight under the high-humidity environment. AQP3 is expressed in the collecting ducts and plays an important role in the urine concentration mechanism (25, 26, 28–30). Severe nephrogenic diabetes insipidus (NDI) phenotype has been observed in AQP 3 knockout mice, and it has been found that mice exhibited remarkable polyuria and polydipsia when given free access to water, suggesting that polydipsia may be a compensatory change (28, 31). Increased water intake was similarly observed in *Aqp3* ^-/-^ mice in this study. Additionally, short-term exposure to a high-humidity environment did not result in pathological changes in the distal colon either in wild-type mice or in *Aqp3^-/-^* mice. Therefore, we hypothesize that short-term exposure to high humidity would not result in significant lesions in intestinal tissues.

AQPs are a family of highly selective transmembrane channels, of which AQP1, AQP3, AQP4, and AQP8 are all expressed in colon (6, 29, 32, 33). AQP1, AQP4 and AQP8 are classical aquaporins and AQP3 is a aquaglyceroporin (17). AQP3, AQP4 and AQP8 are mainly expressed in the colonic epithelium, which can regulate colonic water transport and directly affect intestinal water content and intestinal water metabolism levels (17, 29, 34, 35). Therefore, it has been proposed that AQP3, AQP4 and AQP8 have been the core indicators for studying the mechanism of intestinal water metabolism (6). It has been proposed that AQP1 was primarily found in the kidneys and lungs (36–39). In addition, it was found that the highest amount of AQP1 was found in the small intestine of the gastrointestinal tract, and AQP1 expression correlated with the expression profiles of hypoxia-dependent proteins such as hypoxia induced factor-1α (HIF-1α) and recombinant phosphoglycerate kinase (PGK1) (32). A study has shown that seasonal differences in water metabolism in the intestinal tract were associated with seasonal changes in AQP expression levels, and AQP3 was up-regulated in the colon of mice exposed to 26.2°C and 64.3% of the environment (6). However, the study lacked a control group of mice that housed in a regular environment. In our prior study, an inappropriate diet in conjunction with a high temperature and humidity environment led to the up-regulation of AQP3 and the downregulation of AQP4 in the colon of mice (23). Thus, in this study, we explored the effects of a high-humidity environment on AQP1, AQP3, AQP4 and AQP8 in the colon. The results showed slightly elevated expression levels of colonic AQP1 and AQP8 in a high-humidity environment, and significantly elevated levels of AQP3. Conversely, the expression level of AQP4 was found to be reduced. This suggested that the high-humidity environment mainly affects AQP3 and AQP4 in the colon.

AQP4 acts as an aquaporin that is mainly permeable to water, while AQP3 acts as an aquaglyceroporin that not only contributes to water diffusion, but is also permeable to glycerol, urea, hydrogen peroxide, and some monocarboxylates that can facilitate lactate diffusion (17, 40). AQP3 is the most dominantly expressed AQP in the colon (41). The research has demonstrated that the deletion of AQP4 exerts little effect on the important functions of the colon, including fecal dehydration, fluid absorption, and fluid secretion (24). Over the past decade, AQP3 has received considerable attention due to its critical function in maintaining water transport, cell volume regulation, intestinal permeability, fluid secretion and absorption homeostasis (42, 43). Growing evidence indicates that the expression or alteration of AQP3 in the intestine may be associated with a variety of intestinal disorders, including inflammatory bowel disease, diarrhea, intestinal barrier injury, irritable bowel syndrome, intestinal oxidative stress and autophagy (17). Furthermore, AQP3 plays an important role in the inflammatory processes in human gastrointestinal infectious diseases (18, 44).According to our group’s previous study, high temperature and humidity caused intestinal inflammatory factors elevation and minimal enteritis in mice (8). A study revealed that a low temperature and high humidity environment activates the TLR4/NF-κB pathway through gut microbial disruption (7). According to our previous findings, an inappropriate diet in conjunction with a high temperature and humidity environment led to the upregulation of AQP3, TLR4, and NF-κB p65 protein expression and the downregulation of AQP4 in the mouse colon (23). In the present study, the elevated levels of NF-κB p65, TLR4, and IL-6, as well as the significant increase in colonic AQP3, were observed in the high-humidity environment. Therefore, experiments were conducted using AQP3 knockout mice to ascertain whether the high-humidity environment induced an inflammatory response in the intestines by affecting AQP3. Knockout of AQP3 decreased inflammatory expression in the high-humidity environment. In particular, *Aqp3^-/-^* mice showed higher inflammatory expression in the normal environment than mice in the HH group. We speculate that this is probably attributable to the fact that AQP3 knockdown affects the intestinal barrier integrity (45). Consequently, it can be hypothesized that the high-humidity environment induced an inflammatory response by affecting the key factor AQP3.

Intestinal oxidative stress often occurs with inflammation and disturbs intestinal homeostasis. Many studies on plant extracts have confirmed the involvement of AQP3 in regulating oxidative stress (46–48). Our previous study found that AQP3 overexpression induced high NF-κB expression and intracellular ROS accumulation (49). Furthermore, a high-humidity environment caused hypoxia-related tissue damage in the mitochondria of intestinal tissues (50). Mitochondrial dysfunction is associated with AQP3 overexpression and lipid peroxidation (51). MDA is an oxidation product in organisms and is harmful to organisms. GSH, which can scavenge oxidants, is considered to be the first line of defense against oxidative damage in cells (21). The peroxisome proliferator-activated receptors (PPARs) shape the adaptive and innate immune responses in the lamina propria and subsequent gut homeostasis. Evidence in the scientific literature indicates that PPAR-γ significantly represses the expression of inflammatory genes in trans and interferes with several proinflammatory transcription factors, including NF-κB and TLR4 (52, 53). The results of this paper showed that colonic MDA content was significantly increased and GSH content, as well as the protein content of PPAR-γ, were significantly decreased in a high-humidity environment, whereas the above results were reversed after the knockdown of AQP3.Thus, the high-humidity environment affects the inflammatory response by influencing AQP3, as well as possibly oxidative stress.

In conclusion, current studies suggest that AQP3 may act as an important target for disorders that involve disruption of intestinal fluid homeostasis like diarrhea, constipation, inflammatory bowel disease and irritable bowel syndrome. This study suggested that the high-humidity environment causes increasing intestinal AQP3 expression and generates an inflammatory response. This may affect intestinal diseases. Therefore, this study provided evidence for the effect of climate on intestinal diseases.

## Data Availability

The original contributions presented in the study are included in the article/**Supplementary Material**. Further inquiries can be directed to the corresponding authors.
