# Supplementary material for: Short-term exposure to a high-humidity environment triggers intestinal inflammation via AQP3
Source: Front Immunol. 2025 Jun 18;16:1563602. doi: 10.3389/fimmu.2025.1563602 (PMC12258048; doi:10.3389/fimmu.2025.1563602)
Supplement: Supplementary file 1 [file Table1.docx]

Supplemental Table 1

Primer sequence for RT-qPCR.

| Gene name | Direction | Primer Sequence (5′-3′) |
| --- | --- | --- |
| TLR4 | Forward | ATGGCATGGCTTACACCACC |
|  | Reverse | GAGGCCAATTTTGTCTCCACA |
| NF-κB p65 | Forward | TGCGATTCCGCTATAAATGCG |
|  | Reverse | ACAAGTTCATGTGGATGAGGC |
| IL-6 | Forward | CTGCAAGAGACTTCCATCCAG |
|  | Reverse | AGTGGTATAGACAGGTCTGTTGG |
| AQP1 | Forward | AGGCTTCAATTACCCACTGGA |
|  | Reverse | CTTTGGGCCAGAGTAGCGAT |
| AQP3 | Forward | CCTTGGCATCTTGGTGGCT |
|  | Reverse | AGGAAGCACATTGCGAAGGT |
| AQP4 | Forward | ATCAGCATCGCTAAGTCCGTC |
|  | Reverse | GAGGTGTGACCAGGTAGAGGA |
| AQP8 | Forward | ACACCAATGTGTAGTATGGACCT |
|  | Reverse | TGACCGATAGACATCCGATGAAG |
| GAPDH | Forward | GGTTGTCTCCTGCGACTTCA |
|  | Reverse | TGGTCCAGGGTTTCTTACTCC |
